# Structural Health Monitoring Cost Estimation of a Piezosensorized Aircraft Fuselage

**DOI:** 10.3390/s22051771

**Published:** 2022-02-24

**Authors:** Ilias N. Giannakeas, Zahra Sharif Khodaei, M. H. Ferri Aliabadi

**Affiliations:** Department of Aeronautics, Imperial College London, South Kensington Campus, Exhibition Road, London SW7 2AZ, UK; z.sharif-khodaei@imperial.ac.uk (Z.S.K.); m.h.aliabadi@imperial.ac.uk (M.H.F.A.)

**Keywords:** cost analysis, composite fuselage sensorization, bottom-up cost estimation, added weight, SHM system installation

## Abstract

Guided waves-based SHM systems are of interest in the aeronautic sector due to their lightweight, long interrogation distances, and low power consumption. In this study, a bottom-up framework for the estimation of the initial investment cost $\left(C_{OTC}\right)$ and the added weight $\left(W_{AW}\right)$ associated with the integration of a SHM system to an aircraft is presented. The framework provides a detailed breakdown of the activities and their costs for the sensorization of a structure using a fully wired approach or the adoption of the printed diagnostic film. Additionally, the framework considers the difference between configuring the system for Manual or Remote data acquisition. Based on the case study presented on the sensorization of a regional aircraft composite fuselage, there is a trade-off between $C_{OTC}$ and $W_{AW}$ for the SHM options considered. The Wired–Manual case leads to the lowest $C_{OTC}$ with the highest $W_{AW}$, while the combination of diagnostic film with a Remote system leads to the highest $C_{OTC}$ and the lowest $W_{AW}$. These estimations capture the characteristics of each system and can be integrated into cost–benefit analyses for the final selection of a particular configuration.

## 1. Introduction

Much research has been devoted over the last decades towards the development of Structural Health Monitoring (SHM) techniques that will allow the cost-efficient structural integrity assessment of critical components [1,2,3]. Depending on the structure being assessed, different SHM systems have been proposed in the literature implementing a range of sensors, including accelerometers, strain gauges, optical fibers, and piezoelectric transducers (PZTs) [4,5,6,7].

Guided wave-based SHM (GW-SHM) systems using PZTs have gained recognition for aerospace applications due to their compactness and capability of detecting damage in plate-like structures over long distances as well as interrogating inaccessible areas [8,9,10]. Typical active sensing GW-SHM systems, in particular, employ a network of permanently mounted PZTs to compare the signals from the current (and unknown) structural state with reference signals that have been collected from the pristine structure [3]. During this process, damage-sensitive features are extracted that are used to characterize the structure as healthy or damaged and provide valuable decision-making information. The use of composite materials for structural components in aerospace has increased in popularity due to their stiffness to weight performance [11], and GW-SHM systems have been found capable of detecting barely visible impact damage (BVID) that can significantly reduce their residual strength [12,13]. There are still, however, certain challenges, such as (i) the certification and the assessment of the robustness of the system [8,14,15], (ii) the longevity and durability of the sensors and equipment [16,17,18], and (iii) the quantification of the implementation cost and the added weight [16] that need to be addressed prior to the large-scale adoption of SHM systems.

TAs disclosed from 55 airliners, the operating costs have been reported by the International Air Transport Association (IATA) for the financial year 2013 [19]. It was reported that costs associated with maintenance and overhaul is the third larger portion and amounts approximately for 9.4% of the total costs (with the first being fuel and oil—33.4% and the second the aircraft ownership—10.6%). The introduction of SHM offers the opportunity for a paradigm shift from the current schedule-based practices to condition-based maintenance [20]. These figures indicate that if a SHM system can reduce maintenance costs even by a small margin, it can lead to significant benefits compared to its implementation cost. The final decision of integrating a SHM system for the integrity assessment of a composite structure over the existing schedule-based maintenance strategies will emanate from a cost–benefit evaluation of the proposed system considering the underlying uncertainties and risks.

One of the main impediments in the integration of SHM systems in many industries is the justification of their cost benefit [21]. In aviation, many studies attempt to assess the potential benefits of integrating health monitoring systems in the aircraft maintenance strategy and planning. For instance, the cost–benefit analysis of the implementation and utilization of sensor-based health monitoring strategies is presented in [22]. The logistics and operational costs are also included to highlight the impact on maintenance when health monitoring is considered. A discrete even simulator was proposed in [23] for the planning of maintenance actions based on the prognostics of a health monitoring system. In [24], the cost of integrating and implementing a SHM system was estimated and compared with standard detailed visual inspection that is used as a baseline. In [20], a maintenance cost model was combined with reliability considerations to evaluate the benefits of skipping planned maintenance actions based on the indications of a SHM system. These contributions demonstrate that adopting SHM can reduce the maintenance costs and allow the planning of the maintenance actions, reducing the opportunity costs. Of paramount importance for the integration of a SHM system is the total added weight to the structure as it can influence the operational costs. Based on estimations of fuel consumption increase, the cost of weight (CoW) over the lifecycle of an aircraft is reported in the literature between 1500−2000 $/kg [25,26]. Even in the scenario of a 10% fuselage mass increase due to the integration of SHM, it is estimated in [20] that integrating a SHM system is still beneficial. In [27], the authors presented a cost–benefit analysis considering potential weight saving benefits due to structural sizing reduction when a SHM system is installed. The structural weight savings can offset the SHM added weight and lead to an estimated 1.8% fuel reduction. Sizing reduction was also considered in [26], where fuselage weight savings up to 15% are reported; however, the SHM added weight was not considered. In [24], on the other hand, it was argued that for existing aircraft, the added weight on the structure might have to be compensated by an equivalent reduction in the maximum allowable payload (i.e., fewer passenger seats for a commercial airliner). In such a scenario, it is estimated that the opportunity cost of integrating a SHM system can significantly outweigh the potential benefits.

As reported in [28,29], the majority of the avoidable costs for aircraft manufacturing can be mitigated during the early design stages. It is thus desirable to develop tools to assess the cost impact of SHM adoption during the conceptual design stages following an integrated design philosophy [30]. Many of the contributions mentioned in the previous section consider only specific aspects of the SHM system, or do not include the aircraft level integration, which can affect the accuracy and the transferability of the estimations. In this study, a novel framework is presented for the estimation of the investment cost and added weight that is associated with the acquisition and the aircraft level integration of a SHM system. The estimations are based on a bottom-up approach. The integration of the system is broken down into individual activities that consider the sensorization cost and added weight for each component of the aircraft structure individually. The bottom-up estimations can be realized in a model with a tree structure, combining and propagating the cost and weight estimations of each component to the final structure. Therefore, by modifying the activities, the estimations are not restricted to specific aircraft designs or components. Furthermore, the model is made general and applicable to different scenarios by studying two options for sensorization (wired sensors and diagnostic film), as well as two interrogation/data collection configurations (manual and remote). The activities relating to the integration of each SHM configuration are mapped in detail to identify the associated costs and weights and incorporate into the estimation the characteristics of each option. Such mapping is missing from the literature and can be invaluable if integrated into future cost–benefit studies. Thus, a roadmap is established for the cost and weight estimations of different SHM options, enabling the comparison between them and the identification of the sensitivities of each option. Such mapping of the required activities for different SHM options is currently missing. It is envisaged that the proposed framework will allow design engineers to assess the feasibility of each option during the early design phases of composite aircraft components.

This paper is structured as follows: Section 2 describes the sensorization and interrogation options considered. In Section 3, the bottom-up cost and weight estimation model are described mapping the activities required for each SHM system considered. In Section 4, a case study for the sensorization of a composite fuselage is presented. The case study is based on indicative values, and it is used as an example to compute the cost and added weight and compare the different SHM cases. Section 5 includes a discussion of the results and the considerations required for the selection of a SHM system. Lastly, concluding remarks are included in Section 6.

## 2. SHM System Configurations

It is important to account for the characteristics of each SHM system as they will significantly influence the final cost and added weight. The active GW-SHM system considered for costing is based on a network of surface-mounted PZT wafers. It is considered that sensorization of the structure is carried out using either fully wired sensors or printed diagnostic films. The interrogation of the structure needs to manage which sensor is acting as the actuator. Considering the aircraft level integration of the SHM system, data acquisition can be carried out based on a ‘manual’ or ‘remote’ approach. With the options considered, the following SHM configuration cases are derived: (i) ‘Wired—Manual’, (ii) ‘Printed—Manual’, (iii) ‘Wired—Remote’ and (iv) ‘Printed—Remote’, where the term ‘printed’ is used instead of diagnostic film for brevity. The differences in the SHM options studied are discussed in the following sections.

### 2.1. Sensorization Process

**Figure 1:**
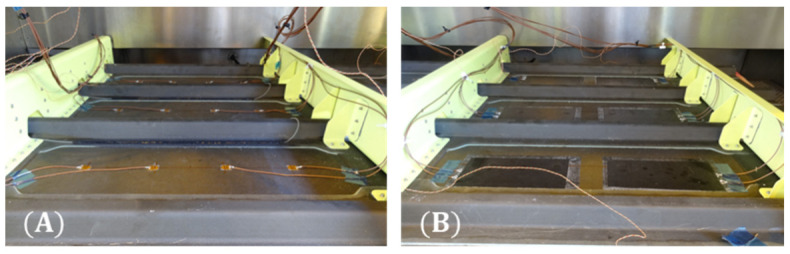
Panel sensorization options (**A**) Fully wired and (**B**) diagnostic film.

The technology used for the SHM system can significantly alter the final cost and weight estimations. Many studies have been conducted to evaluate the robustness of surface-bonded PZTs under different environmental and operational conditions [31,32,33]. Furthermore, the sensorization process adopted for the installation of the sensors must also be considered. Two sensorization options are considered in this study, both of which have been demonstrated to meet the expected mechanical and operational conditions [16,17,33]. These options also have the added benefit of being able to replace faulty sensors without damaging the host structure. Therefore, the installation of DuraAct PZT sensors with the options adopted is assumed to be indicative of the process for realistic applications. Naturally, any other option is applicable by adjusting the respective cost and weight of the sensors in the estimation. The two options are briefly described below and illustrated in Figure 1:

- Wired Sensors: Following this strategy, the PZT wafers are bonded to the surface of the composite using a thermoplastic film [17]. The cables are then soldered onto the PZT electrical contacts directly and routed onto the structure. The application of an additional layer is required to protect and fix the sensors and the cables to avoid becoming projectile during flight.
- Printed Diagnostic Films: This sensorization process aims to deliver a methodology that is scalable for industrial use. The film consists of an array of PZT sensors on an inkjet-printed network of conductive tracks. In this case, the cables are connected to the terminals of the network instead of the sensors, reducing the required cable length [16].

Compared to the wired case, the diagnostic film offers the advantage of improved precision and repeatability for the sensor placement, weight reduction, flexibility to adapt to different geometries, and possibly automation of the sensorization process [16]. However, the extra cost of printing the conductive tracks must be considered. It is noted that the SHM layer [34] and the SMART layer [35] were also tested in [33], while the application of a flexible printed circuit is also presented in [10].

### 2.2. Inspection Approach

Manual SHM systems require the installation of connection ports to the cabin walls that are easily accessible by the inspection personnel [24]. The removal of internal structures can thus be avoided, which reduces downtime. Because all sensor cables must be routed to the nearest connection port, the cable length of each sensor depends on the number and distribution of the connection ports in the aircraft. This necessitates the permanent installation of the connection ports to the aircraft that entails possible structural modifications as well as extra weight.

Contrary to the manual approach, a wireless sensing network (WSN) can be adopted that allows data acquisition to be carried out remotely. This has attracted significant scientific interest as it can streamline the interrogation process and reduce restrictions associated with the positioning of the physical ports, the cost and weight of the wiring, and the transmission of the measured signals over long cable runs [9,36,37]. For aircraft-specific applications, the WSN system must adhere to strict requirements regarding their lightweight construction, performance under different environmental and operational conditions, reliability, and robustness (see [9,38]). An alternative approach to the WSN is to design the SHM system as a subsystem of the Integrated Vehicle Health Management (IVHM) system of the aircraft. Such examples can be found in [27,39].

The WSN system configuration presented in [9] is studied here. The system consists of an array of nodes that are connected directly to the sensor network in their vicinity and a central network coordinator. The nodes are responsible for conducting the active sensing and transmitting the results back to the central coordinator that handles the actuation sequencing and data repository. The specific WSN system is selected as it is developed for aviation applications considering weight and longevity limitations. Furthermore, the interested reader is referred to the contributions presented in [40,41,42] for wireless systems with applications in civil, aerospace, and mechanical infrastructure.

## 3. Materials and Methods: Bottom-Up Cost and Weight Estimation Framework

### 3.1. SHM System Breakdown

Different methodologies have been proposed in the literature for cost estimation studies. According to [28,43], these methods can be classified as analogous, parametric, and bottom-up. The analogous and parametric methods cost drivers and cost estimation relationships are extracted through the analysis of available databases [19,44]. On the other hand, the bottom-up approach maps all steps and identifies the costs of materials, labor, infrastructure, etc., that are required for each step until the completion of the final product [28]. Due to the unavailability of historical data, the bottom-up approach is adopted here.

**Figure 2:**
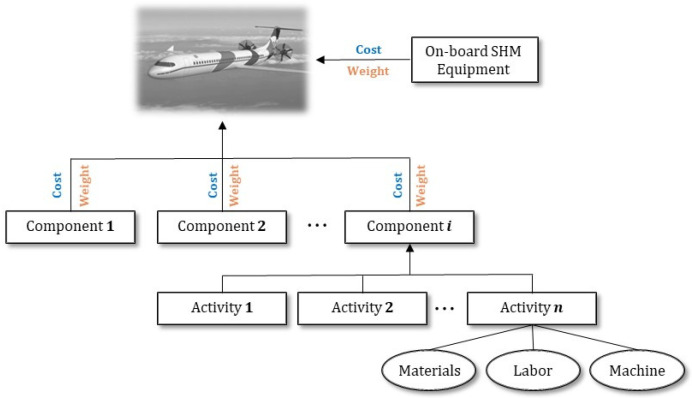
Bottom-up model for the estimation of the total costs and added weight in the complete structure.

The cost and the weight of the SHM system can be broken down into the cost and weight contributions for each individual component of the aircraft by mapping the materials, labor, and equipment used for each activity in a work breakdown structure [28,45] (see Figure 2). The added weight is estimated by accounting for all SHM parts mounted on each component. Because the cost and the weight of the SHM system depend on the configuration selected, particular emphasis is given to mapping all activities that will highlight the characteristics of each one.

Only the direct costs associated with the initial investment required for the integration of the SHM system are considered. These costs are called One-Time Costs ($C_{OTC}$) and include the investment required for the integration of the SHM system and occur at the initial stage of the life-cycle of the system [46]. The costs associated with each part of the craft structure were computed at the component level and propagated to the final structure. Using such an approach, it is also possible to propagate the uncertainty in the parameters to the total estimation. Let q denote the total number of components that are sensorized in the aircraft structure. Then $C_{OTC}$ can be expressed as follows:

(1) $$C_{OTC}=\sum_{1}^{q}C_{Inst,i}+\sum_{1}^{q}C_{Acq,i}+C_{System\;Equip}$$

where $C_{Inst}$ and $C_{Acq}$ are the total installation and acquisition costs for the SHM system, while $C_{System\;Equip}$ is the cost associated with the aircraft level integration of the system.

Similarly, the total added weight to the structure can be broken down as follows:

(2) $$W_{AW}=\sum_{1}^{q}W_{sensors,i}+\sum_{1}^{q}W_{cabling,i}+\sum_{1}^{q}W_{Con,equip,i}+W_{System\;Equip}$$

where $W_{sensors}$ is the sensor weight, $W_{cabling}$ is the cabling weight, $W_{Con,equip}$ is the weight of the connection equipment, and $W_{System\;Equip}$ is the weight associated with the aircraft level integration of the system.

### 3.2. Installation Costs

The costs associated with the installation of the SHM system on the $i^{th}$ component on the structure was computed as follows:

(3) $$C_{Inst,i}=C_{Inst,sensors,i}+C_{Inst,cabling}+C_{Inst,equip,i}+C_{cons,i}$$

where $C_{Inst,sensors,i}$,$\;C_{inst,cabling},$ $C_{Inst,equip,i}$, and $C_{cons}$ denote the cost to install the sensors, the cabling, the onboard equipment, and the installation consumables, respectively.

The sensors are not installed onto the component individually but rather in batches. The total number of sensors required is defined as follows:

(4) $$N_{sensors,d}=N_{batch}N_{per\;batch}$$

where $N_{batch}$ is number of batches for the complete sensorization of the component and $N_{per\;batch}$ is the number of sensors per batch. Assuming that the sensors that failed the QC test are discarded, then:

(5) $$N_{sensors}=\left(1+f_{bond}\right)N_{sensors,d}=N_{sensors,d}+N_{failed}$$

where $f_{bond}$ is the per sensor failure rate of the bonding process. As a worst case, it is assumed that the failed sensors are all from different batches. Thus, the bonding process is repeated for each failed sensor and will be carried out $N_{bond}=N_{batch}+N_{failed}$ times.

**Figure 3:**
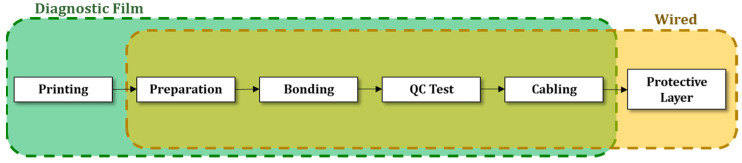
Steps required for the SHM installation.

In the case of the Printed option, if a sensor fails the QC test, the whole diagnostic film is removed. In addition, considering the films that failed the QC test, the total number of prints was estimated as follows:

(6) $$N_{films}=\left(N_{batch}+N_{failed}\right)\left(1+f_{print}\right)$$

where $f_{print}$ is the printing failure rate. The cost to install the sensors on the component is defined by breaking down the sensorization process adopted into individual activities (Figure 3) as follows:

(7) $$C_{Inst,sens,\;i}=\{\begin{array}{ll} C_{prep}+C_{bonding}+C_{QC}+C_{pl} & \mathrm{Wired}\;\mathrm{Option}\\ C_{print}+C_{prep}+C_{bonding}+C_{QC} & \mathrm{Diagnostic}\;\mathrm{Film}\;\mathrm{Option} \end{array}$$

The activities for the sensorization process are briefly described below:

1. Printing: The network of conductive wires are printed using a piezoelectric Dimatix printer. The wires are printed on a 25 μm polyimide (Kapton) film using silver nanoparticle ink. The diagnostic film is then placed in a laboratory oven for sintering the particles.
2. Preparation: The surface of the structure is thoroughly cleaned and sanded to remove contaminants and improve adhesion during the bonding.
3. Bonding: The sensors (or diagnostic film) are bonded to the surface using a thermoplastic film. To achieve a repeatable bonding, the bond area is heated under a vacuum.
4. QC Testing: Electromechanical Impendence measurements (EMI) are recorded for quality control (QC) to assess the integrity of the sensor and the bonding [47]. An advantage of the bonding process is that sensor removal can be performed without damaging the host structure [17].
5. Cabling: After QC testing, the cables are soldered to the PZT sensors (or track terminals in the diagnostic film option) and routed onto the structure.
6. Protective layer: According to Federal Aviation Administration (FAA) and relevant standards for airborne equipment (e.g., RTCA DO-160 [48]), the cables and the sensors must be secured to avoid becoming projectile during flight. In the case of the diagnostic film, this activity is not required.

Following similar bottom-up costing processes from additive manufacturing studies [49,50,51,52], the cost of printing $C_{print}$ is broken down as [52]:

(8) $$C_{print}=C_{CAD}+C_{set-up}+C_{build}+C_{sinter}+C_{QC}^{print}.$$

The cost for the preparation of the conductive track geometry CAD files,$\;C_{CAD}$, is:

(9) $$C_{CAD}=T_{CAD}C_{mh}$$

where $C_{mh}$ is the labor rate and $T_{CAD}$ is the time required for the preparation of the CAD file.

The cost of setting up the printer, $C_{set-up},$ can be computed considering the initial time to set-up the print job $t_{set-up}^{printer}$ and the time required for ink refill $t_{refill}$ using the expression:

(10) $$C_{set-up}=\left(t_{set-up}^{printer}+t_{refill}F_{refill}N_{films}\right)\left(C_{mh}+C_{machine}^{printer}\right)$$

where $F_{refill}$ is a factor that describes the refill frequency.

**Figure 4:**
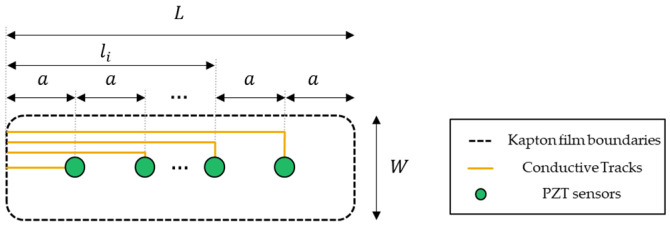
Geometry of the generic diagnostic film configuration for the estimation of the total track length required during printing.

The cost of printing the diagnostic films $C_{build}$ is defined as follows:

(11) $$C_{build}=t_{build}C_{machine}^{printer}N_{films}+C_{ink}V_{ink}N_{films}+1.2C_{subtrate}A_{batch}N_{films}$$

where $t_{build}$ is the printing time, $C_{machine}^{printer}$ is the printer use cost, $C_{ink}$ is the ink cost, $V_{ink}$ is the ink required for each film, $C_{subtrate}$ is the cost of the Kapton film and $A_{batch}$ is the batch area. The factor 1.2 is introduced in Equation (11) to account for substrate waste. The printing time depends on the printing speed, $a_{print}$ and the track length, $l_{tracks}$. The geometry of the conductive tracks are illustrated schematically for a generic diagnostic film configuration in Figure 4.

Unless the exact configuration of the diagnostic film is available, the total track length for the diagnostic film can be estimated using the generic configuration, illustrated in Figure 4. Let a be the distance between the sensors and the edges of the Kapton film. Then, the distance of the ith sensor from the edge is $l_{i}=ia$ and the total track length, $l_{tracks}$, depends on L and $N_{per\;batch}$, as follows:

(12) $$l_{tracks}=1.1\sum_{i}^{N_{per\;batch}}l_{i}=1.1\frac{N_{per\;batch}L}{2}$$

where the factor 1.1 is included in Equation (12) to account for indirect track paths. Then, $t_{build}$ and $V_{ink}$ can be computed as $t_{build}={\left(a_{print}\right)}^{-1}l_{tracks}$ and $V_{ink}=a_{ink}l_{tracks}$, respectively, where $a_{ink}$ is the ink required per track meter. The sintering cost, $C_{sinter}$, was estimated as follows:

(13) $$C_{sinter}=t_{sinter}C_{machine}^{oven}A_{batch}/A_{oven}N_{films}$$

where $t_{sinter}$ is the duration of the sintering phase, $C_{machine}^{oven}$ is the cost of using the oven, and $A_{oven}$ is oven capacity.

Then, $C_{QC}^{print}$ is defined as follows:

(14) $$C_{QC}^{print}=t_{QC}^{print}\left(C_{mh}+C_{machine}^{QC}\right)N_{films}$$

where $t_{QC}^{print}$ is the time required for the QC check of the diagnostic film. Using Equations (8)–(14), the total cost of printing the required diagnostic films is computed.

The cost of preparation, $C_{prep}$, can be estimated as follows:

(15) $$C_{prep}=t_{prep}C_{mh}N_{sensors}$$

where $t_{prep}$ is the person-hours required for surface preparation.

The cost of bonding the sensors of a batch onto the structure could be computed as follows:

(16) $$C_{bonding}=t_{set-up}N_{bond}C_{mh}+C_{machines}^{bonding}t_{bond}N_{bond}+1.2C_{termopl}A_{batch}N_{bond}$$

where $t_{set-up}$ is the person-hours required to apply and seal the vacuum bags over the sensorization area, $C_{machines}^{bonding}$ is the machine cost of the breather and heating blankets, $C_{thermopl}$ is the cost of the thermoplastic film for the bonding, and the factor 1.2 is introduced in the last term of the above equation to account for material waste.

The cost of the bonding QC test can be computed as follows:

(17) $$C_{QC}=t_{QC}\left(C_{mh}+C_{machine}^{QC}\right)+t_{rem}C_{mh}N_{failed}$$

where $t_{QC}$ is the time required to perform the QC inspection of a bonded sensor, $C_{machine}^{QC}$ is the machine use cost, and $t_{rem}$ is the time required to remove a sensor that has failed the QC test.

The cost of the protective layer can be computed as follows:

(18) $$C_{pl}=C_{prot.\;mat}A_{batch}N_{bay}$$

where $C_{prot.\;mat}$ is the cost of the material used as a protective layer. Based on the sensorization option, the terms of $C_{Inst,sens,\;i}$ were computed using Equations (8)–(18).

Next, the cost to install the cabling, $C_{Inst,cabling,i}$, can be estimated as follows:

(19) $$C_{Inst,cabling,i}=t_{Inst,cabling}N_{sensors,i}C_{mh}$$

where $t_{Inst,cabling}$ is the time required for the soldering, labeling, and routing.

The cost for the installation of the component connection equipment, $C_{Inst,equip,i}$, can be computed as follows:

(20) $$C_{Inst,equip,i}=t_{Inst,eqiup}N_{equip,i}C_{mh}$$

where $t_{Inst,eqiup}$ and $N_{equip,i}$ are the time required and the number of the connection equipment required for the specific component. These values depend on the interrogation approach adopted for the specific component.

For the manual case, Bayonet Neill–Concelman (BNC) connectors must be installed at each cable end. The connectors must then be mounted to the designated connection port to allow access to the inspection personnel. For the remote case, $C_{Inst,equip,i}$ refers to the cost of installing the WSN system nodes. Based on the case considered, $C_{Inst,equip,i}$ was computed as follows:

(21) $$C_{Inst,equip,\;i}=\{\begin{array}{ll} t_{Inst,cc}N_{cc,i}C_{mh} & \mathrm{Manual}\;\mathrm{Option}\\ t_{Inst,node}N_{nodes,i}C_{mh} & \mathrm{Remote}\;\mathrm{Option} \end{array}$$

where $t_{Inst,cc}$ and $N_{cc,i}=N_{sensors,d}$ are the installation time and the required number of cable connectors, while $t_{Inst,node}$ and $N_{nodes,i}$ are the installation time and the number of WSN nodes. The number of WSN nodes depends on the channels, $N_{channels}$, each node can accommodate, and the number of nodes was computed as $N_{nodes,i}=ceil\left(N_{sensors,d}/N_{channels}\right)$.

The consumables’ costs were:

(22) $$C_{cons}=N_{sensors,i}C_{cons,sens}$$

where $C_{cons,sens}$ is the cost of consumables per sensor. The consumables include costs, such as cleaning solvents, vacuum bags, etc., that are used during the installation.

### 3.3. Acquisition Costs

The acquisition costs were broken down for each component as follows:

(23) $$C_{Acq,i}=C_{sensors,i}+C_{cabling,i}+C_{equip,i}$$

where $C_{sensors,i}$, $C_{cabling,i}$, $C_{equip,i}$ are the cost for the acquisition of the required sensors, cabling, and connection equipment for the ith component, respectively. These costs were computed as follows:

(24) $$C_{sensors,i}=N_{sensors,i}C_{sensor,HW}$$

(25) $$C_{cabling,i}=C_{cb}l_{cb,i}N_{sensors,d}$$

(26) $$C_{equip,i}=C_{equip}N_{equip,i}.$$

where $C_{sensor,HW}$ is the sensor cost, $l_{cb,i}$ is the cable length required for each sensor, $C_{cb}$ is the cable cost per meter, $C_{equip}$ is the cost of single connection equipment, and $N_{equip,i}$ is the number of the connection equipment, as defined in Equation (20).

The cost of the connection equipment for the different interrogation options can be computed as follows:

(27) $$C_{equip,i}=C_{equip}N_{equip}=\{\begin{array}{ll} C_{cc}N_{cc,i} & \mathrm{Manual}\;\mathrm{Option}\\ C_{node}N_{nodes,i} & \mathrm{Remote}\;\mathrm{Option} \end{array}$$

where $C_{cc}$ and $C_{node}$ are the cost of each cable connector and WSN node, respectively.

### 3.4. SHM System Equipment Costs

The manual inspection requires the acquisition and installation of connection ports, while the remote approach, a network coordinator. These costs are differentiated from $C_{Aqc}$ and $C_{Inst}$ as they refer to the aircraft level integration of the system. A single coordinator is sufficient for the WSN network, while multiple connection ports must be used for the manual approach.

(28) $$\begin{array}{cc} C_{System\;Equip} & =C_{Inst,Sys.\;Eq.}+C_{Acq,Sys.Eq.}\\ & =\{\begin{array}{ll} \left(t_{Inst,Port}C_{mh}+C_{port}\right)N_{ports} & \mathrm{Manual}\;\mathrm{Option}\\ t_{Inst,coord}C_{mh}+C_{coord} & \mathrm{Remote}\;\mathrm{Option} \end{array} \end{array}$$

where $t_{Inst,Port}$ is the time required to install a connection port, $C_{port}$ is the cost of a connection port, $N_{ports}$ is the number of ports, $t_{Inst,coord}$ is the time required for the installation of the coordinator, and $C_{coord}$ is the cost of the network coordinator.

### 3.5. Added Weight Estimation and Cost of Weight

The total added weight of the system is one of the critical factors for the adoption of SHM for commercial applications. The total weight added to each component was computed as follows:

(29) $$W_{Sensors,i}=W_{sensor}N_{sensors,d}+W_{fix}$$

(30) $$W_{cabling,i}=W_{cb}l_{cb,i}$$

(31) $$W_{con,equip,i}=N_{equip,i}W_{equip}$$

where $W_{sensor}$ is the weight of each individual sensor, $W_{cb}$ is the cable weight, $W_{fix}$ is the extra weight added for making sure that any part of the SHM system will not become projectile during a flight, and $W_{equip}$ is the weight of the equipment installed for each component. For remote monitoring, $W_{equip}=W_{node}$ is the weight of each WSN node, while for the manual monitoring, $W_{equip}=W_{cc}$ is the weight of the cable connectors used for each sensor.

In the case of a wired installation, $W_{fix}$ refers to the weight of the protective layer, while for the diagnostic film, to the weight of the printed substrate. Its value can be computed as follows:

(32) $$W_{fix}=\{\begin{array}{ll} A_{batch}/N_{per\;batch}W_{pl} & \mathrm{Wired}\;\mathrm{Option}\\ A_{batch}/N_{per\;batch}W_{subtrate} & \mathrm{Diagnostic}\;\mathrm{Film}\;\mathrm{Option} \end{array}$$

The weight of the equipment for the operation of the SHM system is considered. It is noted that only onboard equipment are considered. Thus, for each interrogation option, $W_{System,Equip}$ was computed as follows:

(33) $$W_{System\;Equip}=\{\begin{array}{ll} N_{ports}W_{port} & \mathrm{Manual}\;\mathrm{Option}\\ W_{coord} & \mathrm{Remote}\;\mathrm{Option} \end{array}$$

where $W_{port}$ is the weight of each port and $W_{coord}$ is the weight of the coordinator.

The added weight due to the integration of the SHM system will increase the fuel costs of the aircraft. Approaches for the computation of the CoW include methods based on the Breguet range equation and the IATA Fuel penalty method [27,53,54]. The latter approach is used here that is based on the statistical analysis of historical data from different airliners. The cost due to added weight is expressed as follows:

(34) $$C_{AW}=C_{fl}\times FF_{incr}\times AvgFLY\times W_{AW}=C_{ef}W_{AW}$$

where $C_{fl}$ is the fuel price per kg, $FF_{incr}$ is the ratio fuel flow increase per kilogram of added weight per flight hour, AvgFLY is the average number of flight hours per year of operation, and $C_{ef}$ is the cost of extra fuel for each additional kilogram added per year.

The $C_{AW}$ can be combined with the initial investment cost $C_{OTC}$ to compute the total cost of deploying the SHM system. Accounting for the net present value of $C_{AW}$ [23], the total cost can be computed as follow:

(35) $$C_{Total}=C_{OTC}+\overset{LcY}{\underset{i=1}{\sum }}\frac{C_{AW}}{{\left(1+r\right)}^{i}}$$

where r=0.02 is the inflation rate.

## 4. Results: Cost and Added Weight Estimation for a Fully Sensorized Smart Fuselage

### 4.1. Case Study Description

As a case study, $C_{OTC}$ and $W_{AW}$ are estimated for the sensorization of a regional aircraft fuselage. The example used is based on the ongoing activities of the SHERLOC project for the development of a fully sensorized smart composite fuselage. This case study is used as an example to allow the comparison between the different SHM cases considered. Although the values considered for this case study are only indicative, they allow for the comparison between the SHM configurations considered.

Multiple sensors are placed inside each bay of the fuselage to create a network of sensors and maximize the probability of detecting flaws. In total, the fuselage consists of $N_{bays}=\left(2\pi RL_{fslg}\right)/\left(L_{bay}W_{bay}\right)=1034$ bays and each bay is a batch for the installation (i.e., $N_{bays}=N_{batch}$). For each bay, the batch area value was assumed as $A_{batch}=L\times W=0.75\;L_{bay}\times 0.2=0.093{\;m}^{2}$. With $N_{per\;bay}=N_{per\;batch}=4,$ a total of $N_{sensors,d}=4136$ was required. For all estimations relating to labor activities, the rate $C_{mh}=£95/h$ was used. The inputs assumed to represent a regional aircraft fuselage are summarized in Table 1. The resulting network density was approximately $26\;\mathrm{sensors}/m^{2}$. Such values are close to similar studies in the literature (e.g., $33\;\mathrm{sensors}/m^{2}$ in [24]).

The implementation of the presented cost and weight model requires the estimation of all parameters discussed in Section 3. Naturally, there will be uncertainty with each value due to fluctuations in market prices, transportation, storage, personnel experience, equipment used, etc. Following the best practices for cost estimation [55,56,57], the uncertainty in each parameter can be considered by fitting a triangular distribution to the upper, lower, and most likely values. The assumed values are reported in Table 2. This allowed the uncertainties to be propagated to the final estimations and evaluate the sensitivity to each parameter.

Whenever possible, information from order sheets was used (e.g., DuraAct sensors, cabling, BNC connectors). In such cases, a 10% variation from the expected value was assigned. The cost and the weight of the connection ports, the WSN nodes, and the network coordinator, on the other hand, was highly uncertain as such devices are still under development. For the WSN node, it is noted in [9] that each node is expected to weigh around 80 g and cost £450. The cost was indicative of the prototype build, and the final price could be lower for mass production, while in the weight estimation, the power supply and the casing were not considered. Similar products weigh between 120 g–700 g [39,58]. It was assumed that each node would cost £500, weigh 0.5 kg, and service $N_{channels}=24$ sensors [9,39]. The upper and lower values were set as such to capture the range reported in the literature. The cost and the weight of the connection ports and the Network coordinator were assumed, and their uncertainty was reflected in the limits used. The data for the installation process were based on observations from on-going sensorization activities, as well as values reported in the relevant literature (see [11,17]). The expected values were extracted based on the performance of experienced personnel. Therefore, a 10% variation was assumed for the lower value and 20% for the upper. The values used for the printing of the diagnostic films were based on a Dimatix DMP 2850 printer, while the machine use costs, were assumed values. Although an attempt was made to report values in Table 2 that are realistic, they were based on empirical observations. These values were used as a case study to set a basis for the comparison between the different SHM configurations.

**Figure 5:**
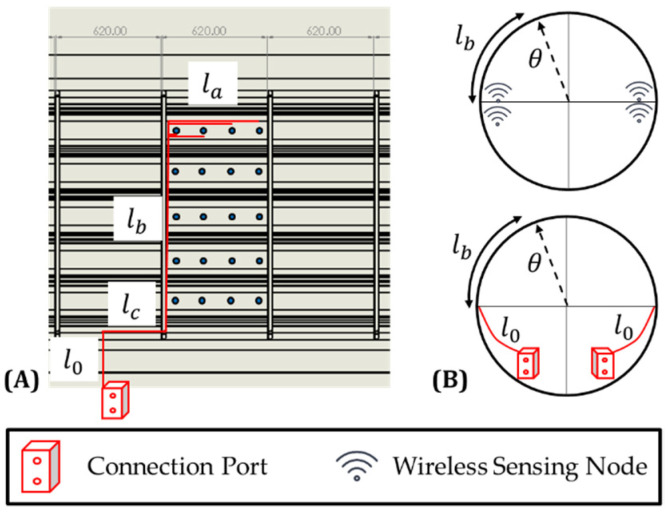
Illustration of the cable routing for the aircraft fuselage (**A**) side view and (**B**) cross-se view, indicating the location of the connection ports and the WSN nodes.

Of utmost importance was the accurate estimation of the cable length required for each sensor. The length of the cable depends on both the sensorization and interrogation method adopted and had a profound impact on $W_{AW}$. The total cable length required for each sensor can be defined as follows:

(36) $${\hat{l}}_{cb}=\left({\hat{l}}_{a}+{\hat{l}}_{b}+{\hat{l}}_{c}+{\hat{l}}_{0}\right)k_{cb}.$$

where each term of Equation (36) illustrated schematically in Figure 5A, and $k_{cb}=1.2$ is a factor to account for indirect paths and cable slack [27].

The distribution of the sensors inside the bay was assumed uniform. If the diagnostic film approach is followed, no cabling is required, and ${\hat{l}}_{a}$ was computed for each case as follows:

(37) $${\hat{l}}_{a}=\{\begin{array}{ll} \frac{\sum_{i=1}^{n}l_{a,i}}{N_{per\;bay}}=\frac{\sum_{1}^{n}ia}{N_{per\;bay}}=\frac{L_{bay}}{2} & \mathrm{Wired}\;\mathrm{Option}\\ 0 & \mathrm{Diagnostic}\;\mathrm{Film}\;\mathrm{Option} \end{array}$$

where $l_{a,i}$ is the distance between the ith sensor and the fuselage frame, and $a={\left(N_{per\;bay}+1\right)}^{-1}L_{bay}$.

Both the connection ports and the WSN nodes were assumed to be placed symmetrically on both sides of the aircraft (see Figure 5B). In the case of WSN nodes specifically, where multiple nodes were needed to service the sensors, they could be grouped together due to their miniaturized size [9]. Then, ${\hat{l}}_{b}$ was computed as follows:

(38) $${\hat{l}}_{b}=\frac{2}{\pi R}R^{2}\int_{0}^{\pi /2}\theta d\theta =\frac{\pi R}{4}.$$

Lastly, ${\hat{l}}_{c}$ and ${\hat{l}}_{0}$ were defined. These terms were only relevant for the manual interrogation approach. The definition of ${\hat{l}}_{c}$ depended on the number and distribution of connection ports in the aircraft. It was assumed that both the distance between two frames ($L_{bay})$ and the longitudinal distance between two ports ($L_{ports})$ were uniform. Then, the cables from each frame of the fuselage were connected to the closest connection port:

(39) $$l_{c,i}=\min\left[\left(floor\left(\frac{iL_{frame}}{L_{port}}\right)L_{port}-x_{i}\right),\left(ceil\left(\frac{iL_{frame}}{L_{port}}\right)L_{port}-x_{i}\right)\right]$$

Thus, ${\hat{l}}_{c}$ was computed as follows:

(40) $${\hat{l}}_{c}=\{\begin{array}{ll} l_{c,i}/N_{ports} & \mathrm{Manual}\;\mathrm{Option}\\ 0 & \mathrm{Remote}\;\mathrm{Option} \end{array}$$

Finally, ${\hat{l}}_{0}$ was taken as a constant value to represent a possible offset of the connection port with respect to the fuselage wall. It was assumed:

(41) $${\hat{l}}_{0}=\{\begin{array}{ll} 0.4\;m & \mathrm{Wired}\;\mathrm{Option}\\ 0 & \mathrm{Diagnostic}\;\mathrm{Film}\;\mathrm{Option} \end{array}$$

The illustrations in Figure 5A,B provide a localized view of the cabling routing in a portion of the fuselage. Using the above assumptions for the cabling, it was possible to estimate the total cable length required for the whole fuselage.

Given the specific choices for the SHM system, the cable length for each sensor was computed using Equations (36)–(41).

### 4.2. Cost and Added Weight for the SHM System Integration

**Figure 6:**
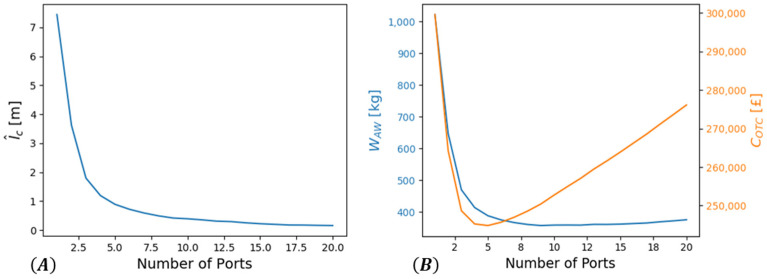
(**A**) Variation of ${\hat{l}}_{c}$ and (**B**) $W_{AW}$ and $C_{OTC}$ with respect to the number of connection ports installed.

Using the data Table 2, $C_{OTC}$ and $W_{AW}$ were computed for each SHM system case outlined in Section 2. As a first step, ${\hat{l}}_{cb}$ must be computed for each case. The maximum value corresponded to the Wired–Manual, while the minimum to the Printed–Remote case since ${\hat{l}}_{a}={\hat{l}}_{c}={\hat{l}}_{0}=0$. For the Manual systems, ${\hat{l}}_{cb}$ depended on the number of connection ports through ${\hat{l}}_{c}$ (see Figure 6A). It was observed that there was a significant variation in both $W_{AW}$ and $C_{OTC}$ based on the number of connection ports installed, as illustrated in Figure 6B. The final location and number of the connection ports were influenced by design considerations, but this observation is indicative of the need to consider the aircraft level integration of the SHM system instead of looking locally at each component separately when the manual option is considered. It is noted that as a simplification, the ports were assumed to be uniformly distributed along the fuselage length.

Using Equation (35), the number of ports can be optimized solving for $\underset{N_{ports}}{\mathrm{argmin}}\;C_{Total}\left(N_{ports}\right)$. For the estimation of $C_{AW}$, the values $C_{fl}=0.6\;£/\mathrm{kg}$ [59], $FF_{incr}=0.04$, AvgFLY=3000 fh/year and LcY=20 years were used that were reasonable for a regional aircraft [54]. This leads to the optimal value $N_{ports}=9$, which is used hereafter for both manual cases.

**Figure 7:**
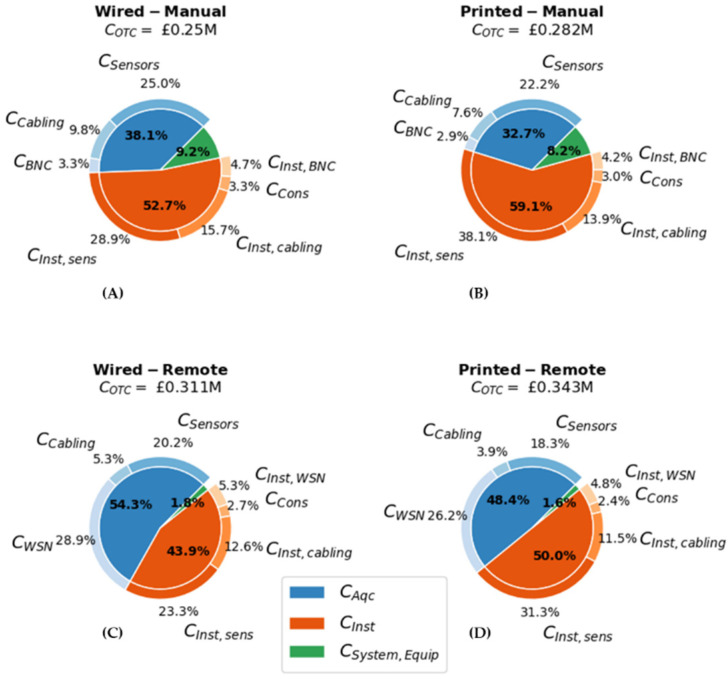
Breakdown of the acquisition $\left(C_{Aqc}\right)$, installation $\left(C_{Inst}\right),$ and system equipment $\left(C_{System,Equip}\right)$ costs for the (**A**) Wired–Manual, (**B**) Printed–Manual, (**C**) Wired–Remote and (**D**) Printed–Remote SHM system configurations.

**Figure 8:**
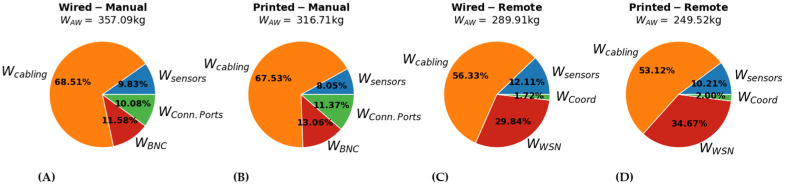
Breakdown of $W_{AW}$ for the (**A**) Wired–Manual, (**B**) Printed–Manual, (**C**) Wired–Remote, and (**D**) Printed–Remote SHM system configurations.

Using the expected values from Table 2, the breakdown of $C_{OTC}$ and $W_{AW}$ is reported in Figure 7 and Figure 8, respectively. In the Remote cases $C_{System\;Equip}$, i.e., the cost of procuring and installing the network coordinator, was much less significant than the Manual case that included the procurement and installation of the connection ports. This further illustrates the need to consider the aircraft level integration of the SHM system, as it may otherwise lead to the underestimation of $C_{OTC}$ for the Manual cases. Although $C_{cabling}$ and $C_{System\;Equip}$ were reduced for the Remote case, this reduction was outweighed by increased costs for $C_{WSN}$ which became the main contributor for $C_{Aqc}$. In total, shifting from a Manual to a Remote SHM system can increase $C_{OTC}$ by approximately £57,000.

Adopting the diagnostic film increased $C_{Inst,sensors}$ due to the printing costs. It was estimated that $C_{print}=£35,000$ for the complete sensorization of the fuselage. Accounting for the £3000 reduction in $C_{cabling}$ due to the reduced cable length, shifting from a Wired to a Printed system increased $C_{OTC}$ by approximately £32,000.

The added weight is one of the main constraints when designing airborne equipment, as it can significantly impact the operational costs of the aircraft. In all cases, the cabling required for the connection of the sensor network was the main contributor to $W_{AW}$. Reducing ${\hat{l}}_{cb}$ can, therefore, impact significantly $W_{AW}$. Shifting from a Wired to a Printed system, ${\hat{l}}_{cb}$ was reduced by 0.31 m. This reduced $W_{Cabling}$ by approximately 30.8 kg. Additionally, $W_{AW}$ was further reduced by avoiding the requirement of adding an extra protective layer for fixing the sensors and the cabling. This translated to a further reduction of 7.7 kg. In total, shifting from a Wired to a Printed case reduced $W_{AW}$ by approximately 38.5 kg.

The choice between a Manual or the Remote system significantly affected $W_{AW}$ as it was possible to install the WSN nodes close to the sensor. Comparing the Remote with the Manual cases, ${\hat{l}}_{cb}$ was reduced by approximately 0.98 m, which reduced $W_{cabling}$ by 81.3 kg. At the same time, however,$\;W_{con,equip}$ and $W_{System\;Equip}$ were increased. For the Manual case $W_{BNC}+W_{System,equip}=77.36\;\mathrm{kg},$ while for the Remote case $W_{WSN}+W_{System,equip}=91.5\;\mathrm{kg},$ leading to a total benefit of 67.18 kg. As with the estimation of $C_{OTC}$, $W_{AW}$ would be underestimated for the Manual case if the aircraft level integration was not considered.

There are uncertainties associated with the estimation of the $C_{OTC}$ and $W_{AW}$. According to the best practices for cost estimation [55,56,57], a triangular distribution can be fitted when upper, lower, and most likely values are available (e.g., in an expert’s opinion). To account for bias, it was assumed that the lower and upper bound corresponded to the 90% probability region [55,56]. If historical data are available, more appropriate distributions can be fitted. Following [60], triangular distributions were assigned to the model parameters using the ranges reported in Table 2 and samples were then drawn using Sobol’s sequences to explore the input space.

**Figure 9:**
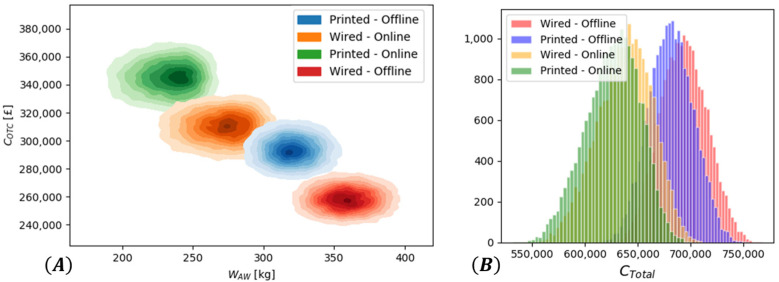
(**A**) Density plots of $C_{OTC}$ and $W_{AW}$ (**B**) Estimated $C_{Total}$ for the different SHM system cases.

The $C_{OTC}$ and $W_{AW}$ were estimated for each sample drawn, and the resulting densities are plotted in Figure 9A. There was a clear trade-off between $C_{OTC}$ and $W_{AW}$ that must be taken into consideration by the system integrators during the selection of a particular option. Despite the increase in $C_{OTC}$ associated with the Printed cases, $C_{Total}$ (Figure 9B) could be reduced due to the reduction in $W_{AW}$. This was observed in both the Manual and Remote options.

**Figure 10:**
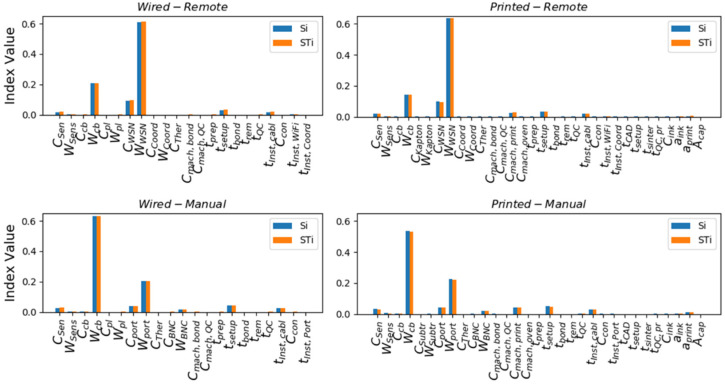
Sensitivity Index $\left(S_{i}\right)$ and the Total Effect Index $\left(S_{Ti}\right)$ for the parameters influencing $C_{Total}$.

To assess the sensitivity of $C_{Total}$ on the uncertainty assigned to each of the model parameters, Sobol’s variance-based global sensitivity analysis method was used, following [61,62]. For each of the model parameters, the Sensitivity Index $\left(S_{i}\right)$ and the Total Effect Index $\left(S_{Ti}\right)$ were computed based on 100,000 samples. The resulting indexes are summarized in Figure 10. In all cases, it was observed that $\sum S_{i}\approx 1$ and $S_{Ti}\approx S_{i}$, indicating the problem is perfectly additive, and there are no significant interactions between the parameters.

In the Manual cases, it was observed that the variability in $C_{Total}$ was mainly influenced by the uncertainty in $W_{cb}$ and $W_{port}$, with $W_{cb}$ being the main contributor. This is expected due to the longer cable lengths required. Comparing the Wired–Manual and the Printed–Manual cases, the influence of $W_{cb}$ was reduced, while for $W_{port}$, it was increased. In the Remote cases, ${\hat{l}}_{cb}$ was further decreased. The variability of $C_{Total}$ was mainly attributed to the uncertainty in $W_{WSN}$, while using Printed instead of Wired sensors further reduced the influence of $W_{cb}$. These observations are indicative of the influence of the uncertainties associated with the computation of $W_{AW}$ in the overall cost of integrating an SHM system.

## 5. Discussion

The framework presented in this study is aimed at providing estimations for $C_{OTC}$ and $W_{AW}$ and identifying the influence of uncertainties in the inputs of the model. The trade-off between $C_{OTC}$ and $W_{AW}$ between the SHM cases presented can prove invaluable for system integrators that must adhere to budgetary and weight limits constraints. The selection of a particular SHM system and the ultimate decision for shifting from the traditional NDT inspection to SHM will require a life-cycle cost–benefit assessment considering all potential risks and the characteristics of each option. The SHM technology used will also significantly alter the final cost and weight estimations. Other technologies to the ones adopted here can be considered by making the necessary modifications in the activities included in the framework.

The diagnostic film offers the opportunity to scale-up production and automate installation, making it attractive for large-scale applications and incentivize further research. The added weight to the structure was reduced by reducing the cabling requirements; however, the extra cost of printing the conductive tracks must be included in the estimations.

The Manual option, although it had a lower $C_{OTC}$, is expected to have higher inspection costs since the inspection personnel will have to physically connect the interrogation equipment to each connection port. It is also expected that the Remote configuration will also reduce the duration of each inspection since the interrogation process can be automated, decreasing opportunity costs due to unavailability [24]. Furthermore, integration of a Remote system enables the on-demand and regular interrogation of the structure that can lead to the adoption of true condition-based maintenance strategies.

On the other hand, there are uncertainties regarding the robustness and durability of the equipment for the Remote case as they must adhere to the specifications of RTCA/DO-160 [48]. Equipment failures (such as sensor faults [63] or WSN nodes malfunction) can impair damage detectability and impose significant costs for the maintenance of the SHM system itself [14,16]. Certification costs have not been included in the above estimations as the focus of the present study was to establish a framework for the comparison of different SHM approaches. Although the estimation of the certification costs can be very case-specific, such estimation can be invaluable for OEMs.

The efficacy of the power supply required for the WSN network must also be assessed. Depending on the inspection frequency, the batteries for the system presented in [9] can last over 4 years; however, to avoid maintenance actions due to battery degradation, other SHM systems propose the connection with the aircraft avionics for the power supply [27]. In such a scenario, the power cabling can be accounted for in $C_{OTC}$ and $W_{AW}$ by modifying Equations (28) and (33), respectively. Here, the WSN nodes were assumed to be grouped at two locations of the fuselage cross-section (see Figure 5) to allow easy maintenance access. Further reductions in $W_{cabling}$ are possible if the nodes are uniformly distributed.

**Figure 11:**
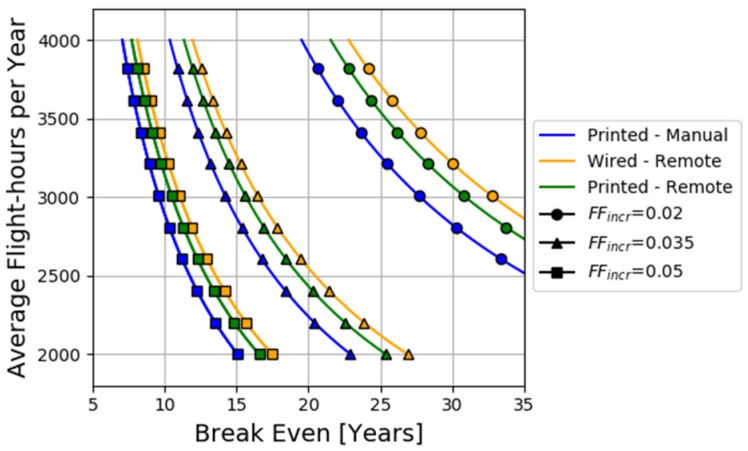
Estimation of the break-even point in years for different combinations of AvgFLY and $FF_{incr}$.

The final decision is influenced by the operational characteristics of the aircraft. The values used for $C_{Total}$ in Figure 9B led to an estimated cost of £1440 per kg added. Although this value is close to similar estimations in the literature ([25,26]), changes in the operation of the aircraft will affect the balance between $C_{OTC}$ and $W_{AW}$. Using the Wired–Manual case as a benchmark, the years in operation required to compensate for the increased $C_{OTC}$ with the reduction in $W_{AW}$ are plotted in Figure 11. The ranges 2000≤AvgFLY≤4000 and $0.02\leq FF_{incr}\leq 0.05$ were assumed based on data reported in [54]. In cases of low AvgFLY and $FF_{incr}$, the reduction in $W_{AW}$ might not be capable of justifying the increase in $C_{OTC}$.

It is obvious that the final selection of a SHM system will depend on a holistic cost–benefit analysis that takes into consideration all aforementioned aspects of the SHM life cycle. Nevertheless, the present approach offers the framework to make estimations on $C_{OTC}$ and $W_{AW}$ for different options, considering the characteristics of each. It is noted that different aircraft types and components can be assessed within the presented framework. The characteristics of each aircraft are encoded through its geometry that affects, for instance, the number of sensors required and the cable length and the added weight cost parameters in Equations (34) and (35).

## 6. Conclusions

In this study, a bottom-up framework was proposed for the estimation of the total weight $\left(W_{AW}\right)$ and the initial investment cost $\left(C_{OTC}\right)$ required for a GW-SHM system. The framework aimed at providing a structured roadmap based on a generic definition of the activities required for the acquisition and integration of the SHM system, allowing the comparison between different sensorization and interrogation options.

In total, two sensorization and two interrogation options were studied. These approaches were combined to produce the Wired–Manual, Printed–Manual, Wired–Remote, and Printed–Remote SHM systems cases. The activities associated with the integration of each option were mapped and formulated. Such formulations were missing from the literature, and this way, the characteristics of each system were included in the estimations.

The case study of a piezosensorized composite fuselage was used to facilitate the comparison between the different options. The estimations made were based on indicative data from ongoing sensorization activities for the development of a fully sensorized smart fuselage. The results indicated that there is a trade-off between $C_{OTC}$ and $W_{AW}$, depending on the system configuration considered. Using the Wired–Manual case as a baseline, the Printed–Manual, the Wired–Remote and Printed–Remote cases increased $C_{OTC}$ by 12.8%, 24.2%, and 37%, while $W_{AW}$ was decreased by 11.3%, 18.8%, and 30.1%, respectively. By converting $W_{AW}$ into added weight cost ($C_{AW}$), it was demonstrated that the lifecycle characteristics of the aircraft (flight cycle per years, expected service life, etc.) could indicate if the weight reduction justifies the increase in the initial investment cost required. For instance, despite the increased added weight associated with the Wired–Manual configuration, this option might be beneficial to aircraft with low utilization compared to the other configurations. Such comparisons can be invaluable for system integrators that have to adhere to several constraints regarding investment budget or allowable weight increase and for system developers to set targets their system must achieve.

Furthermore, the sensitivity of each SHM option was studied. The bottom-up approach adopted requires the identification and quantification of model parameters that might not be readily available to the designer. On the other hand, following an analogous or parametric philosophy [28] requires the use of existing databases that might not be available. Here, triangular distributions were constructed based on assumed lower, expected, and upper limits for the inputs. If more accurate information is available, more appropriate distributions can be fitted. The variability of $C_{total}$ is mainly influenced by the parameters associated with the weight of the SHM system. Design consideration can significantly alter the estimations as the location, distribution, and number of connection ports or WSN nodes that can be installed will be affected. This further highlights the need to consider the SHM system during the early design phases of the aircraft.

## Figures and Tables

**Table 1:** Inputs for the regional aircraft fuselage.

Variable	Description	Units	Value
$L_{fslg}$	Fuselage Length	m	15
R	Fuselage Diameter	m	1.7
$L_{bay}$	Bay Length	m	0.62
$W_{bay}$	Bay Arc Length	m	0.25
$A_{batch}$	Batch Area	$m^{2}$	0.093
$C_{mh}$	Labour Rate	£/h	95
$N_{per\;bay}$	Number of Sensors per Bay		4

**Table 2:** Cost and weight data for the SHM system components.

SHM Costs and Weights					
Variable	Description	Units	Lower	Expected	Upper
$C_{sensor,HW}$	DuraAct Sensor Cost	£	45	50	55
$W_{sensor}$	DuraAct Sensor Weight	kg	0.0045	0.005	0.0055
$C_{cb}$	Coaxial Cable Cost	£/m	1.8	2	2.2
$W_{cb}$	Coaxial Cable Weight	kg/m	0.018	0.02	0.022
$C_{cc}$	BNC Connector Cost	£	1.8	2	2.2
$W_{cc}$	BNC Connector Weight	kg	0.009	0.01	0.011
$C_{substrate}$	Kapton film Cost	$£/m^{2}$	33.3	37	40.7
$W_{substrate}$	Kapton film Weight	$\mathrm{kg}/m^{2}$	0.045	0.05	0.055
$C_{pl}$	Protective layer Cost	$£/m^{2}$	0.9	1	1.1
$W_{pl}$	Protective layer Weight	$\mathrm{kg}/m^{2}$	0.135	0.15	0.0165
$C_{port}$	Connection Port Cost	£	700	1000	1500
$W_{port}$	Connection Port Weight	kg	1.5	2	3
$C_{node}$	WSN Node Cost	£	400	500	550
$W_{node}$	WSN Node Weight	kg	0.25	0.5	0.55
$C_{coord}$	Network Coordinator Cost	£	4500	5000	5500
$W_{coord}$	Network Coordinator Weight	kg	4.5	5	5.5
$C_{termopl}$	Thermoplastic Film Cost	$£/m^{2}$	18	20	22
**SHM System Installation**					
**Variable**	**Description**	**Units**	**Lower**	**Expected**	**Upper**
$t_{prep}$	Surface preparation	h	0.018	0.02	0.024
$t_{set-up}$	Set-up for the sensor bonding	h	0.45	0.5	0.6
$t_{bond}$	Curing duration	h	0.5	0.5	0.55
$t_{rem}$	Removal of failed sensors	h	0.045	0.05	0.06
$t_{QC}$	QC check duration	h	0.018	0.02	0.024
$t_{Inst,cabling}$	Cabling installation	h	0.09	0.1	0.12
$t_{Inst,Port}$	Connection port installation	h	2.7	3	3.6
$t_{Inst,node}$	WSN node installation	h	0.9	1	1.2
$t_{Inst,coord}$	Coordinator installation	h	4.5	5	6
$C_{cons,sens}$	Installation consumables	£	1.8	2	2.2
$f_{rate}$	Bonding failure rate		0.01		
**Printing Information**					
**Variable**	**Description**	**Units**	**Lower**	**Expected**	**Upper**
$t_{CAD}$	CAD Geometry preparation	h	2.7	3	3.3
$t_{set-up}^{printer}$	Ink-jet printer setup	h	0.9	1	1.2
$t_{sinter}$	Sintering duration	h	0.5	0.5	0.6
$t_{QC}$	QC check duration	h	0.045	0.05	0.06
$C_{ink}$	Silver Particle Ink	£/mL	21.15	23.5	25.85
$a_{ink}$	Ink use rate	mL/m	0.36	0.4	0.48
$a_{print}$	Print Speed	m/h	1	1.25	1.375
$A_{oven}$	Oven Capacity	$m^{2}$	0.9	1	1.2
$f_{rate}$	Printing failure rate	h	0.01		
**Machine Use Costs**					
**Variable**	**Description**	**Units**	**Lower**	**Expected**	**Upper**
$C_{machine}^{bonding}$	Bonding Equipment cost	£/h	4	5	8
$C_{machine}^{printer}$	Printer use cost	£/h	15	20	30
$C_{machine}^{oven}$	Sintering oven use cost	£/h	18	20	22
$C_{machine}^{QC}$	QC equipment use cost	£/h	4	5	8

## Data Availability

Not applicable.
